# Exploring the gut microbiota’s crucial role in acute pancreatitis and the novel therapeutic potential of derived extracellular vesicles

**DOI:** 10.3389/fphar.2024.1437894

**Published:** 2024-07-26

**Authors:** Yijie Li, Jie Li, Sen Li, Shumin Zhou, Jiahua Yang, Ke Xu, Yafeng Chen

**Affiliations:** ^1^ Putuo Hospital, Shanghai University of Traditional Chinese Medicine, Shanghai, China; ^2^ Wenzhou Institute of Shanghai University, Wenzhou, China; ^3^ Institute of Translational Medicine, Shanghai University, Shanghai, China

**Keywords:** acute pancreatitis, gut microbiota, gut microbiota-derived extracellular vesicles, pathogenesis, complications, complications treatment

## Abstract

During acute pancreatitis, intestinal permeability increases due to intestinal motility dysfunction, microcirculatory disorders, and ischemia-reperfusion injury, and disturbances in the intestinal flora make bacterial translocation easier, which consequently leads to local or systemic complications such as pancreatic and peripancreatic necrotic infections, acute lung injury, systemic inflammatory response syndrome, and multiple organ dysfunction syndrome. Therefore, adjusting intestinal ecosystem balance may be a promising approach to control local and systemic complications of acute pancreatitis. In this paper, we reviewed the causes and manifestations of intestinal flora disorders during acute pancreatitis and their complications, focused on the reduction of acute pancreatitis and its complications by adjusting the intestinal microbial balance, and innovatively proposed the treatment of acute pancreatitis and its complications by gut microbiota-derived extracellular vesicles.

## 1 Introduction

Acute pancreatitis (AP) is one of the most common acute abdominal conditions in clinical practice, and its incidence is increasing year by year (Li et al., 2020a). The pathology of AP is characterised by the activation of pancreatic enzymes in the alveolar cells, which leads to self-digestion of pancreatic tissues and the release of various pro-inflammatory cytokines, such as TNF-α, IL-1, IL-6, and IL-8, resulting in pancreatic inflammation (Wang et al., 2009). Although the majority of APs result in only self-limiting mild disease, there will still be a proportion of patients whose disease progresses to severe systemic inflammatory response syndrome (SIRS) and/or multiple organ dysfunction syndrome (MODS), and these may result in patients requiring long-term Intensive care unit (ICU) care (Lerch et al., 1993). Not only does it aggravate the physical pain of the patient, but it also adds to the financial burden, there are no effective drugs for the treatment or prevention of AP.

The human gut micro-ecosystem includes thousands of species of bacteria and trillions of microbial cells, accounting for the vast majority of the total number of microorganisms in the human body. It is an essential and complex micro-ecosystem of the human body (Yang et al., 2022). The human gastrointestinal flora can be roughly divided into three main groups according to their different functions within the host: physiological bacteria, which account for more than 90% of the total and have nutritional and immunomodulatory roles; opportunistic bacteria, which are pathogenic in the case of low immune resistance or the abuse of antibiotics; and pathogenic bacteria, which are fewer and less likely to invade (Cen et al., 2018a). Gut microbiome also regarded as a hidden “metabolic organ” that has an important impact on our metabolism, physiology, nutrition and immune function (Guinane and Cotter, 2013). The intestinal microbiota participates in a variety of important physiological functions, such as digestion and absorption, energy metabolism, immunomodulation and intestinal mucosal defence through the regulation of neural, immune and endocrine functions (Sekirov et al., 2010). Research has shown that the gut microbiome has evolved alongside us; therefore, any change in the microbiome can have significant consequences, both beneficial and harmful (Ley et al., 2008). In recent years, with the development of macrogenomic research, relevant studies have found that dysbiosis of intestinal flora is associated with gastrointestinal diseases, such as inflammatory bowel disease (Fischer, 2019), irritable bowel syndrome (Wang et al., 2019), colon cancer (Zeller et al., 2014), etc. The role of the gut microbiota in the host is not only affecting gastrointestinal disorders, but also other distant organs such as the lungs, heart and liver. Once the balance between host and gut microbiota is disrupted, it leads to imbalances in the gut microbiome, which ultimately leads to diseases such as intestinal (Yu et al., 2017), neurological (Stilling et al., 2015), cardiovascular (Wang et al., 2012a) and immune disorders (Kamada et al., 2013).

Extracellular vesicles (EVs) are nano-scale membrane vesicles with a phospholipid bilayer structure that are secreted by almost all cells. Currently, EVs have been isolated from animals (Wu and Liu, 2018), plants (Zhuang et al., 2015) and microorganisms (Briaud and Carroll, 2020). In recent years, EVs have become key players in communication between the gut microbiota and mammals (Tao and Guo, 2018). In mammals, EVs are mainly classified as exosomes, microvesicles, and apoptotic vesicles based on their source of production and size. Exosomes are endocytosis-derived EVs with a size range of 30–150 nm, whereas microvesicles range from 100–1,000 nm in diameter and are formed by the plasma membrane; apoptotic vesicles range from 50 to 5,000 nm and originate from cells undergoing apoptosis (Doyle and Wang, 2019). Microbial-derived EVs contain nucleic acids, proteins, and lipids that play critical roles in host metabolism and health (Liu et al., 2018a). Microbe-host interactions mediated by EVs can lead to a variety of responses, with potential roles as virulence factor delivery vectors or modulators of inflammatory responses (Kim et al., 2013).

In particular, gut microbiota-derived EVs have been implicated in a variety of host functions, including immune system regulation and cancer suppression, in addition to which it has been suggested that these EVs might play a role in gut-brain axis regulation, as they could reach the central nervous system and modulate brain functions (Macia et al., 2019; Haas-Neill and Forsythe, 2020). In addition to showing beneficial effects and their contribution to host homeostasis, alterations in EVs of intestinal microbial origin have been associated with the progression of several diseases, such as human immunodeficiency virus (HIV), inflammatory bowel disease, cancer treatment-induced intestinal mucositis (Tulkens et al., 2020) and Alzheimer’s disease (Lee et al., 2020).

Recent studies suggest a relationship between the pancreatitis and the gut microbiota. Anatomically, the pancreas is connected to the gastrointestinal tract via the pancreatic duct, and pancreatic exocrine function is an important host factor influencing the composition and diversity of intestinal flora (Frost et al., 2019). In addition, gut microbiota dysbiosis affects the severity of pancreatitis (Zhu et al., 2019). Most of the bacteria found to cause necrotising infections of pancreatic tissue are from the gut microbiota, such as *Escherichia coli* and *Enterococci* (Ammori, 2003). During the pathological development of pancreatitis, tight junction proteins are altered, intestinal permeability is increased and the intestinal barrier is damaged (Ryan et al., 1993; Sonika et al., 2017). When the intestinal barrier is disrupted, intestinal bacteria may migrate to the blood or other tissues, further increasing the progression of AP (Li et al., 2020b). In conclusion, the development of AP is accompanied by disturbances in the intestinal flora, bacterial translocation, and impaired intestinal barrier function (Horst et al., 2009), suggesting that there is a significant link between AP and the intestinal microbiota.

This paper offers a detailed overview of the dynamic changes in gut microbiota that occur during the development, progression, and treatment of AP. It sheds light on the mechanisms that underlie these changes in gut microbiota and the complications that may arise from them. Additionally, the paper summarizes therapeutic approaches that aim to modulate the gut microbiota as a means of mitigating the impact of AP. Notably, it introduces the innovative concept of utilizing gut microbiota-derived extracellular vesicles as a potential therapeutic strategy for managing complications associated with AP, offering a promising direction for treatment.

## 2 Dysbiosis of the intestinal flora in acute pancreatitis

With the development of 16S rRNA gene sequencing and high-throughput sequencing techniques, Several studies have shown significant changes in the diversity of the gut microbiome and the relative abundance of specific bacterial taxa during AP. Alterations in the gut microbiome were detected in patients with AP as evidenced by a decrease in *Bifidobacteria* and an increase in *Enterobacteriaceae* and *Enterococci*, but the relative abundance of *Lactobacillus* appeared to be unchanged (Tan et al., 2015). Chen et al. applied that at phyla level, *Saccharibacteria* and *Tenericutes* decreased significantly. At genus level, *Escherichia-Shigella* and *Phascolarctobacterium* increased, while *Candidatus Saccharimonas*, *Prevotellaceae UCG-001*, *Lachnospiraceae UCG-001*, *Ruminiclostridium five* and *Ruminococcaceae UCG-008* decreased significantly (Chen et al., 2017). The study found that fecal samples from AP patients contained more *Bacteroidetes* and *Proteobacteria* and fewer *Firmicutes* and *Actinobacteria* comparing to healthy controls (Zhang et al., 2018). Another research conducted by Zhu et al. found that changes in the gut microbiota in AP included an increase in the relative abundance of opportunistic pathogens such as *Escherichia* and *Shigella*, and a decrease in the relative abundance of beneficial genera, *Prevotella_9*, *Faecalibacterium*, *Blautia* and *Lachnospiraceae* (Zhu et al., 2019). Supplementary Table S1 summarises the significant changes in the composition of the gut microbiota in patients and in animal studies with mild, moderate and severe AP through the use of 16S rRNA gene sequencing and high-throughput sequencing techniques. In conclusion, at the phylum level, AP patients showed increased abundance of *Bacteroidetes* and *Proteobacteria* and decreased abundance of *Firmicutes* and *Actinobacteria*. At the genus level, AP patients and animals showed a significant increase in the abundance of *Escherichia*, *Enterococcus*, *Enterobacter*, *Streptococcus*, etc., and a decrease in *Bacteroides*, *Bifidobacterium*, and *Blautia*, etc.

### 2.1 The mechanism of intestinal microbiotic dysbiosis in acute pancreatitis

#### 2.1.1 Intestinal motility dysfunction

Gastrointestinal motility dysfunction often occurs during the progression of AP (Wang et al., 2003). Zhou et al. observed in a rat model of acute necrotizing pancreatitis (ANP) that the development of intestinal dyskinesia was associated with dysfunction of interstitial cells of Cajal (ICC) and myenteric neurons. (Zhou et al., 2011). Additionally, intestinal motility dysfunction may be attributed to factors such as the neuroendocrine system, hypoxia-ischemia, ischemia-reperfusion injury (IRI), inflammatory mediators, and Cajal cells. (Eshraghian and Eshraghian, 2011). Disturbances in the migrating motor complex (MMC) during ANP can potentially lead to the overgrowth of duodenal Gram-negative and anaerobic microflora. (Van Felius et al., 2003). Furthermore, impaired gut motility can result in the accumulation of harmful substances and inhibit the growth of probiotics, which are crucial for maintaining gut health.

#### 2.1.2 Microcirculation disturbance and ischemia–reperfusion injury

The intestinal mucosa is highly sensitive to ischaemia and hypoxia. Ischaemia causes varying degrees of local tissue damage, which is further exacerbated by reperfusion. In AP, large amounts of cytokines and vasoactive agents are produced, that leads to reduced perfusion of the intestinal tissue and consequently to impaired microcirculation in the intestinal mucosa, and IRI (Camargo et al., 2014). Wang et al. demonstrated that intestinal IRI results in alterations in colonic microbiota, specifically promoting the overgrowth of *E. coli* and *Prevotella oralis*. (Wang et al., 2012b).

#### 2.1.3 Cell apoptosis, necroptosis and intestinal epithelial tight junction destruction

Intestinal epithelial cells, the immune system, and the microbiota interact to maintain normal intestinal homeostasis and barrier integrity. Death of one of these intestinal epithelial cells may lead to increased intestinal permeability and intestinal barrier dysfunction (Subramanian et al., 2020).

In the context of severe acute pancreatitis (SAP), the body undergoes severe oxidative stress, which can lead to excessive activation of the caspase-3 apoptosis pathway, thereby accelerating the apoptosis of intestinal epithelial cells and exacerbating intestinal damage. Concurrently, endoplasmic reticulum stress also plays a significant role in SAP. It activates the tumor necrosis factor receptor 1 (TNFR-1)-mediated receptor interaction, subsequently triggering the activation of the serine/threonine-protein kinase 1 (RIPK1)/RIPK3/mixed lineage kinase domain-like protein (MLKL) signaling pathway. This activation results in the production of the effector protein CCAAT/enhancer-binding protein (CHOP), which then activates the Puma protein, ultimately inducing necrosis in patients with AP. (Wang et al., 2016). Impairment of intestinal barrier function, including increased intestinal permeability, decreased expression of tight junction-associated molecular proteins such as ZO-1 and E-cadherin, and decreased microbiota abundance, was found in a mouse model of SAP (Jin et al., 2022).

#### 2.1.4 Necrotic material in the abdominal cavity

Pancreatitis ascites is caused by persistent leakage of peritoneal pancreatic secretions due to pancreatic duct injury (He et al., 2019). Many studies have shown that pancreatitis-associated ascites (PAAF) contains high levels of toxic substances, including tumour necrosis factors, interleukins and endotoxin, which induce necrosis of pancreatic tissues and exacerbates the inflammatory response (Liu et al., 2018b). A clinical study found that the positivity rate of bacterial infection increased with the course of the disease by bacterial culture of ascites from patients with AP, but nothing has been said about the origin of the ascites bacteria (Ni et al., 2023)

#### 2.1.5 Dystrophy and immune dysregulation

SAP patients are at risk of malnutrition due to the severity of their condition, which affects their ability to eat on their own, slows down gastrointestinal peristalsis and reduces the absorption of nutrients, coupled with metabolic disorders in the organism, rapid energy consumption, and a disruption of the balance between nutrients ingested and nutrients consumed. Plasma Endothelin levels are increased in the early stages of SAP, whereas excessive release of the anti-inflammatory cytokines IL-10 and IL-4 in the late stages leads to immune dysregulation, which plays an important role in infections and organ dysfunction in SAP patients (Shen et al., 2011).

#### 2.1.6 Oxidative stress

Oxidative stress is an imbalance of oxidants and antioxidants in the body, leading to an infiltration of inflammatory cells, which promotes the production and accumulation of reactive oxygen species (ROS) in the cells, ultimately leading to oxidative stress (Hybertson et al., 2011). It was found that malondialdehyde (MDA) and xanthine oxidase (XO) levels were elevated while important antioxidants such as superoxide dismutase (SOD) and glutathione were decreased in experiments with SAP mice. This suggests that severe oxidative stress and activation of the caspase-3 pathway occur in intestinal mucosal cells during SAP, thereby exacerbating intestinal barrier dysfunction (Tian et al., 2013). When SAP occurs, inflammatory cells infiltrate the gut and induce oxidative stress (Yao et al., 2019). Inflammation promotes an oxidative state, which enhances the abundance of *Actinobacteria* and *Aspergillus* (Lazar et al., 2019). Reducing inflammation and oxidative stress has a positive effect on shaping the stability of the gut microbiota (Darnaud et al., 2018).

### 2.2 The impact of intestinal microbiotic dysbiosis on the progress of acute pancreatitis

**FIGURE 1:**
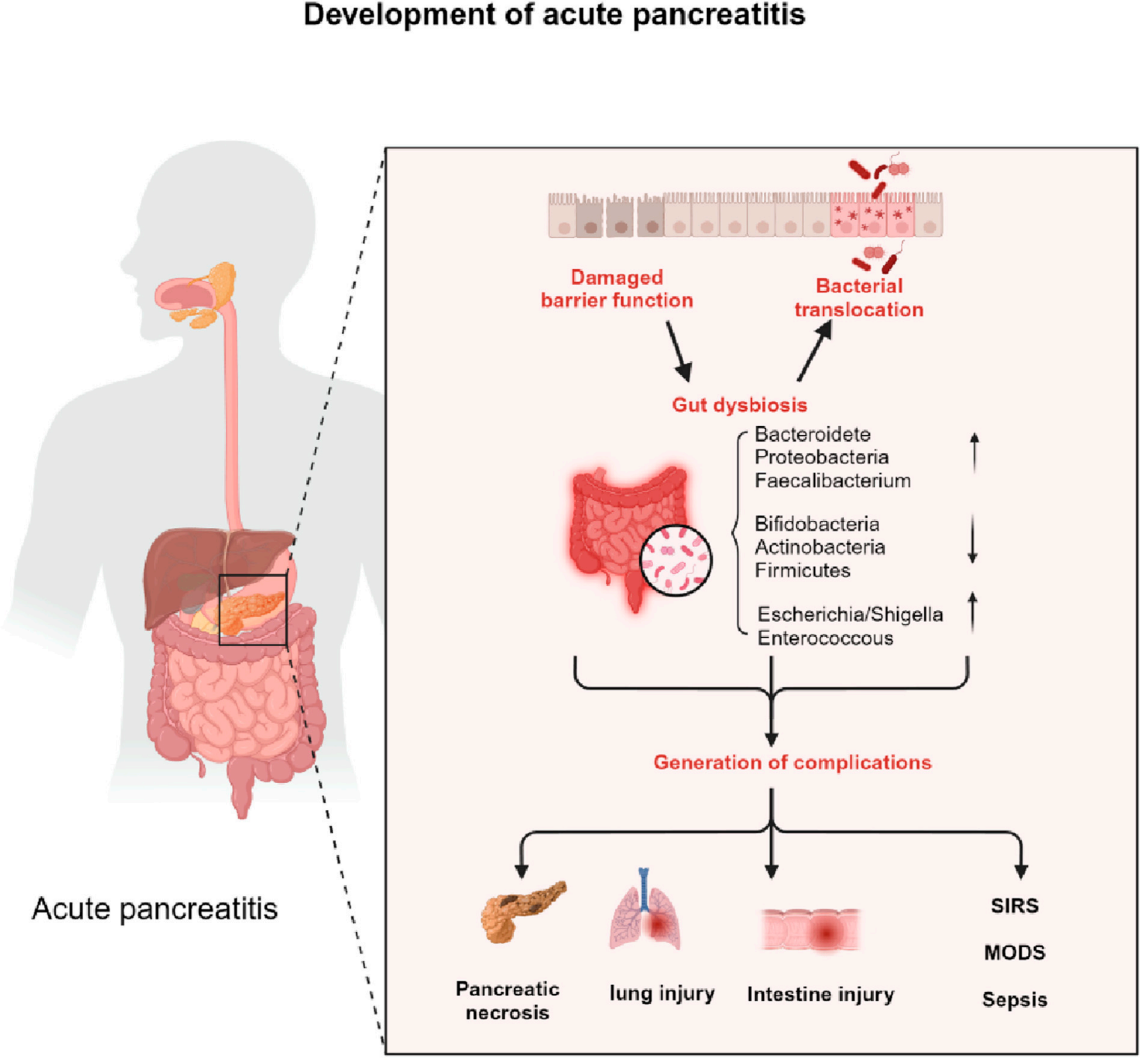
The impact of intestinal microbiotic dysbiosis on the progress of AP. Acute pancreatitis due to microcirculatory disorders, ischaemia-reperfusion injury, and release of inflammatory mediators etc lead to functional impairment of the intestinal mucosa, followed by dysbiosis of the intestinal flora and translocation of intestinal bacteria causing local or systemic complications.

Acute pancreatitis due to microcirculatory disorders, ischaemia-reperfusion injury, and release of inflammatory mediators, etc., lead to functional impairment of the intestinal mucosa as shown in Figure 1, followed by dysbiosis of the intestinal flora and translocation of intestinal bacteria causing local or systemic complications (Ammori et al., 1999).

#### 2.2.1 Local complications

Secondary infections of the pancreas and peripancreatic tissues have been reported in 80% of SAP patients (Al-Omran et al., 2010a). The pathogens of secondary infections are all of enteric origin, mostly *E. coli*, *enterococci* and certain anaerobes (Ge et al., 2020). During the initial phase of SAP, cellular detachment and apoptosis of the villi occur in the intestinal mucosa, the height of the villi and the thickness of the intestinal mucosa are significantly reduced, the tight junctions between the cells are disrupted, the structure and function of the intestinal mucosa are impaired, the intestinal flora is shifted, and a localised necrotic tissue infection may occur (Narayanan et al., 2021). For example, necrotizing pancreatitis (NP) is characterized by further necrosis of the pancreas and peripancreas. Specifically, in imaging, this condition is evident by a lack of enhancement in the pancreatic parenchyma or the presence of peripancreatic necrosis, such as acute necrotic collections or walled-off necrosis. (Maatman et al., 2020). It is closely related to bacterial infections (Bugiantella et al., 2016).

#### 2.2.2 Systemic complications

AP, a common acute abdominal condition, often leads to multi-organ failure, including the bowel. Bowel dysfunction occurs in the early stages of AP and is associated with poor prognosis, such as infectious complications. After the onset of infection, severe stress stimulates deterioration of the patient’s condition and an increased incidence of systemic complications (Ge et al., 2020). Disruption of tight junction proteins, apoptosis of intestinal epithelial cells, and intestinal damage in the form of villous denudation, lamina propria disintegration, and ulceration were detected in the induced AP rat model (Lutgendorff et al., 2009a). Acute lung injury (ALI) is a common systemic complication of AP, which is primarily characterized by progressive hypoxemia and respiratory distress. In severe cases, it can develop into acute respiratory distress syndrome (ARDS) or even MODS, adversely affecting human health (Zhou et al., 2021). The pathology of SAP-induced acute lung injury is characterised by marked pulmonary oedema and alveolar septal damage, along with the presence of leukocyte, erythrocyte and neutrophil retention and aggregation in the alveolar lumen (Liu et al., 2014). Impaired intestinal barrier, increased intestinal permeability and disturbed intestinal microbiota in AP patients allow endotoxin and bacterial translocation, inflammatory factors and activated trypsin to pass through the intestinal-lymphatic-pulmonary axis, which further triggers and promotes lung disease in AP patients (Shah and Rana, 2020). In a retrospective study of 149 patients with AP, Liang et al. used hydrogen breath test to detect changes in gut bacteria in the early stage of AP, and found that gut bacterial overgrowth in the early stage of SAP was associated with ARDS (Liang et al., 2021). In a subsequent study, Hu et al. similarly demonstrated that alterations in gut flora in the early stages of AP were associated with the development of ARDS, and further suggested that *Enterobacteriaceae* and *Escherichia-Shigella* could serve as important predictive biomarkers for AP-ARDS (Hu et al., 2023). Molecular techniques based on 16S rDNA, probed bacterial DNA in the peripheral blood of patients with mild and severe AP and showed a positive correlation between *bacteraemia* and disease severity, with the susceptible organisms being mainly composed of opportunistic pathogens originating from the gut, including *E. coli*, *Shigella fowleri*, *Enterobacteriaceae bacterium*, *Acinetobacter lwoffi*i, *Bacillus coagulans* and *Enterococcus faecalis*. These pathogenic organisms invading the peripheral blood may have a significant impact on systemic infections, septic complications and even MODS in AP (Li et al., 2013). Yu et al. found that the species composition of the gut microbiota in 80 patients with AP of varying severity showed that *Bacteroides anomalies*, *Escherichia Shigella* and *Enterococcus* were the predominant gut microbial species in MAP, MSAP and SAP, respectively. It is suggested that this decrease in the abundance of beneficial bacteria and increase in harmful bacteria aggravated the severity of AP and increased the risk of infection and MODS (Yu et al., 2020). Activation of trypsin during AP induces local inflammation, with necrotic tissues and cells releasing large amounts of inflammatory mediators, accompanied by the release of inflammatory mediators, chemokines, adhesion molecules, oxygen-free radicals, PAFs, and endothelin, which further progresses local inflammation towards SIRS (Ge et al., 2020). The inflammatory response is exacerbated by SAP affecting the functional integrity of the intestinal barrier, as well as toxic substances such as endotoxins and PLA through the somatic circulation and mesenteric lymphatic pathways, leading to more severe SIRS (Leal-Lopes et al., 2015).

## 3 Treatment of complications by regulating the gut microbiota

**FIGURE 2:**
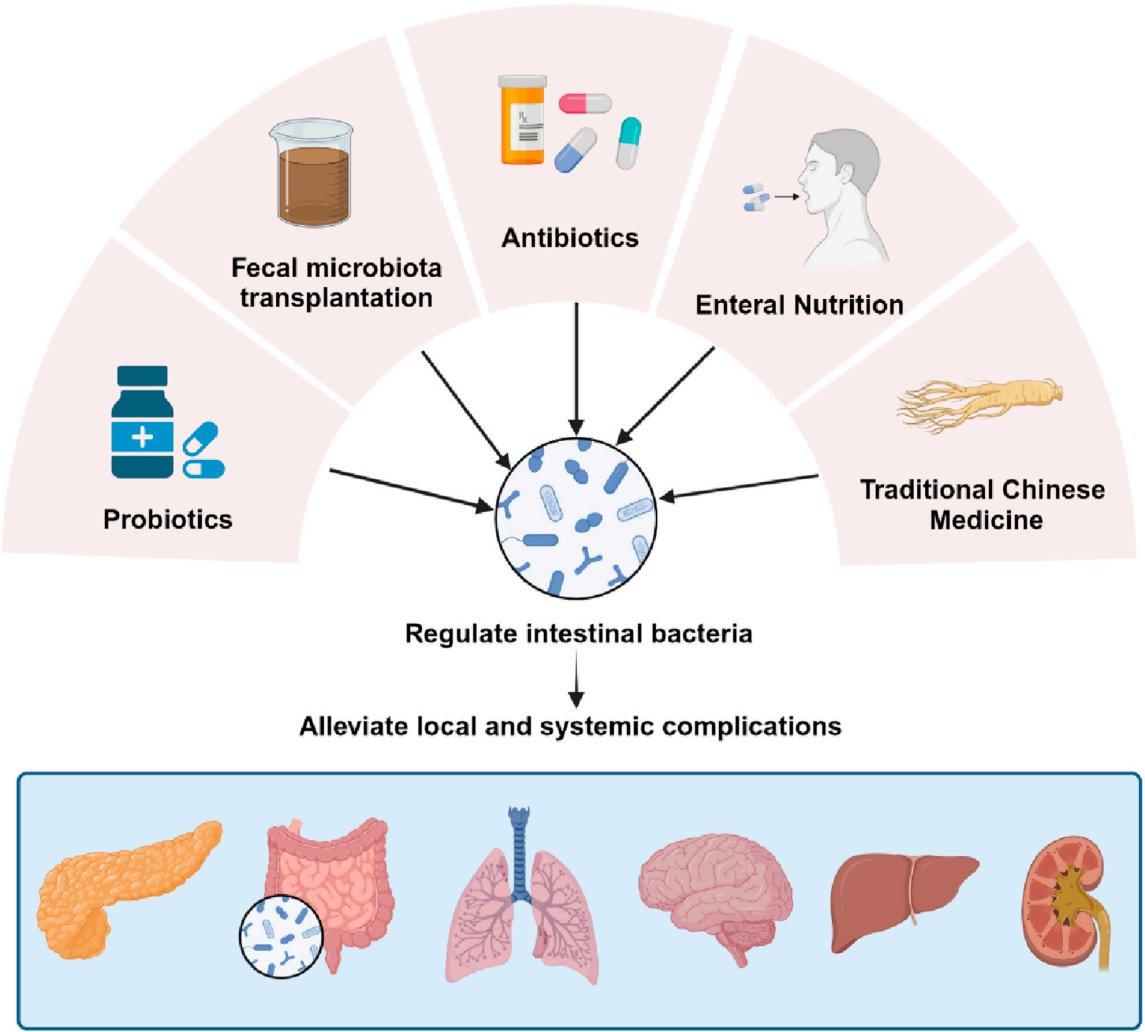
Treatment of complications by regulating the gut microbiota. Modulating the gut microbiota through probiotic and prebiotic supplementation, FMT, antibiotic therapy, EN, and TCM interventions, thereby indirectly protecting the gut barrier and mitigating bacterial translocation and inflammatory recurrence during AP.

The gut microbiota has served as a promising therapeutic target for the treatment of AP and its associated complications due to the interaction of the gut microbiota with AP. By modulating the gut microbiota, as shown in Figure 2, the intestinal barrier can be indirectly protected, alleviating bacterial translocation and inflammatory flare-ups during AP. This chapter provides a comprehensive review of clinical and basic research on various treatment methods for acute pancreatitis, encompassing the supplementation of probiotics and prebiotics, fecal microbiota transplantation (FMT), antibiotic therapy, early enteral nutrition (EN), and traditional Chinese medicine (TCM) interventions. Supplementary Table S2 has been included to visually demonstrate the research progress of each treatment method.

### 3.1 Probiotics and prebiotics

Probiotics are officially defined as “live microorganisms which when administered in adequate amounts confer a health benefit on the host”. Probiotics are often seen as a way of restoring or improving microbiota dysbiosis and are effective treatments for a variety of gastrointestinal conditions, including *Clostridioides*. *Difficile* infection, antibiotic-associated diarrhea, and acute infectious diarrhea. *Lactobacillus* species are used as probiotics, *Lactobacillus salivarius* is effective against *Listeria* infection, and *Lactobacillus reuteri* prevents antibiotic-associated diarrhoea. In addition, *Bifidobacterium animalis* has been shown to prevent infections in infants and *E. coli Nissle* and is an effective treatment for Crohn’s disease and inflammatory bowel disease (Langdon et al., 2016). In recent years, the role of probiotics in restoring intestinal dysbiosis and improving and preventing intestinal mucosal damage has been extensively studied. Probiotics function in human health in four main ways: (1) Specific strains such as *Lactobacillus* and *Bifidobacterium* act as microbial barriers that directly exclude or inhibit pathogens. (2) Probiotics can enhance the intestinal barrier through a variety of pathways that involve induction of mucus and adenosine monophosphate (AMP) production, enhancement of tight junctions and attenuation of epithelial cell apoptosis. (3) Probiotics can modulate the local or systemic immune system. (4) Probiotics may influence gut motility by increasing short-chain fatty acids (SCFAs) production and interacting with the enteric nervous system (Zhu et al., 2021). Probiotics were found to reduce intestinal bacterial overgrowth, protect the intestinal barrier and reduce bacterial translocation, which helped reduce the incidence of infectious complications in AP rats (Van Minnen et al., 2007). In a meta-analysis of the effects of probiotics on AP (Hooijmans et al., 2012), it was concluded that supplementation with probiotics improves pancreatic pathological damage and reduces the incidence of bacterial translocation to the pancreas and mesenteric lymph nodes (MLN). The probiotic-treated rats showed no signs of diarrhoea or loss of appetite, decreased epithelial cell apoptosis, decreased mucosal lipid peroxidation and reduced AP-induced increase in *E. coli* passaging. The above mentioned reduction in intestinal barrier function and oxidative stress damage was the result of probiotic pretreatment with AP, the mechanism of which may be related to mucosal glutathione biosynthesis. Glutathione-dependent glutathione release was also found to be abundant in *Bifidobacterium bifidum*, and *Bifidobacterium lactis* and *Lactobacillus acidophilus*. Since it is worth noting that the probiotics used in this experiment were multi-species combinations of probiotic strains, the effects found were dependent on the combination of bacteria applied, and the effects against each individual strain and the possible synergistic effects of this particular probiotic combination need to be elucidated by further studies (Lutgendorff et al., 2009a). A recent study (Werawatganon et al., 2023) demonstrated that serum amylase levels were significantly reduced in AP mice treated with either a single strain of probiotic or a mixed-strain probiotic, with the effect being more noticeable in the mixed-strain probiotic group in particular. Olah et al. (Oláh et al., 2002) reported inhibitory effect of *Lactobacillus* on sepsis in patients with pancreatitis. *Bacteraemia* and length of hospitalization (LOH) were significantly lower in the group receiving synthroid compared to the placebo group, however the role of synthroid in reducing septic and non-septic complications in patients with moderate and severe acute pancreatitis was not significant (Rohith et al., 2023). *Lactobacillus royale* repairs TNF-induced intestinal epithelial damage, protects the intestinal mucosal barrier from bacterial infection, inhibits bacterial translocation, improves intestinal inflammation, and reduces intestinal damage (Wu et al., 2020). A double-blind clinical trial (Wan et al., 2021) showed that supplementation with *Bacillus subtilis* and *E. faecalis* resulted in significantly shorter length of hospitalization and abdominal pain in patients with AP compared to the placebo group. Commensal *Bifidobacterium* regulate macrophages and inflammatory immune signalling pathways to limit local and systemic inflammatory immune responses (Li et al., 2022). *Lactulose* intervention effectively increased the diversity and improved the structure of the gut microbiota, as evidenced by enhanced α-diversity of gut flora; increased the abundance of the hydrogen-producing bacteria *Prevotellaceae* and *Rikenellaceae*, the probiotic bacteria *Bifidobacteriaceae* and *Lactobacillaceae*, and the mucus-degrading bacteria *Akkermansia* and *Helicobacter*; and decreased the abundance of harmful bacteria *Desulfovibrioaceae* and branched-chain SCFAs (Zhai et al., 2018). This reduces the incidence of complications due to disturbances in the intestinal microbiota caused by AP. In a randomised trial of patients with moderate-to-severe complicated with intestinal dysfunction, serum cytokine levels and intestinal permeability index were found to be reduced after lactulose treatment, and the lactulose group was enriched with the potentially beneficial genus *Bifidobacterium*, suggesting that lactulose treatment significantly improves intestinal function and corrects intestinal dysfunction in patients with AP (Wang et al., 2023). Chitosan oligosaccharides (COS) administration reduced inflammatory infiltration and oxidative stress, restored intestinal injury and barrier dysfunction, and increased the proportion of the probiotic *Akkermansia*, while the pathogenic bacteria *Escherichia–Shigella* and *Enterococcus* almost disappeared in SAP mice. The study suggests that COS administration treats SAP by reducing oxidative stress and restoring intestinal homeostasis (Mei et al., 2021).

### 3.2 Fecal microbiota transplantation

FMT is a technique whereby gut microbiota extracted from the feces of a healthy donor is transplanted into the gastrointestinal tract of a patient. The aim is to restore the normal microbiota and thus cure the disease (Vindigni and Surawicz, 2017). Mostly, this therapy is used to treat gastrointestinal disorders caused by the activity of pathogenic or conditionally pathogenic microorganisms, however, a growing number of recent studies have reported the use of faecal microbiota transplantation for the treatment of diseases such as metabolic syndrome, diabetes, cancer and Parkinson’s disease (Antushevich, 2020). FMT can restore intestinal microecology to reduce oxidative stress in ALI rats, and its regulatory mechanism may be related to the TLR4/NF-kB signalling pathway (Tang et al., 2021). Notably, a study by Ding et al. (Ding et al., 2022) noted that FMT not only does not help with intra-abdominal pressure (IAP) and infectious complications in AP patients, but also negatively affects the intestinal barrier. The feasibility of using FMT to improve bowel function in AP patients needs to be supported by more evidence from clinical randomised trials.

### 3.3 Antibiotics

Antibiotics are commonly used in clinical practice to prophylactically inhibit the growth of pathogenic bacteria in patients with AP and to reduce secondary AP infection complications. The combination of vancomycin, neomycin and polymyxin b reduces intestinal bacterial translocation to the pancreas and inhibits the inflammatory response to AP (Jia et al., 2020). Noteworthy, meropenem pretreatment accelerated mortality in AP mice, which may be related to the translocation of multidrug resistant (MDR) strains, mainly *Enterococcus gallinarum*, into the blood stream (Soares et al., 2017). According to the recent World Society of Emergency Surgery (WSES) guidelines for the management of SAP, prophylactic use of antibiotics in patients with AP is not associated with a reduction in mortality or morbidity (Leppäniemi et al., 2019). Therefore, antibiotics are no longer recommended as routine treatment for patients with AP. In addition to this, the selection of broad-spectrum antibiotics should take into account the bacteriological changes in the disease and its pharmacokinetics (Wittekamp et al., 2020).

### 3.4 Enteral nutrition

EN treatment of AP patients at the appropriate time point is not only beneficial for the recovery of normal physiological functions, but also reduces the rate of bacterial translocation, can maintain the functional and structural integrity of the intestinal mucosa, and can significantly reduce AP morbidity and mortality (Al-Omran et al., 2010a). EN has a positive effect on reducing the incidence of MODS and death by maintaining the functional integrity of the intestinal mucosa, reducing bacterial translocation and intestinal infections, promoting gastrointestinal peristalsis, and increasing perfused blood flow to the intestinal mucosa (Liu et al., 2023). It was found that after enteral feeding of *Lactobacillus plantarum*, it has a role in restoring the integrity of the intestinal barrier and microbiota, while reducing the incidence of sepsis in patients with AP (Qin et al., 2008). Wang et al. found that the percentage of pancreatic sepsis, multiple organ dysfunction syndrome and mortality were significantly lower in the EN group, and further lower in the EN + ecoimmunonutrition (EIN) group than in the Parenteral nutrition (PN) group. EN plays an important role in the treatment of SAP by decreasing the expression of endotoxin, TNF-alpha, and IL-6, as well as bacterial translocation, and by enhancing the expression of IL-10, and the combination of EIN and EN is more effective than EN alone in the treatment (Wang et al., 2013). Enteral nutrition is important in the treatment of AP, and current research emphasises enteral nutrition in combination with other therapies for AP. In the meta-analysis of rhubarb combined with early EN for the treatment of SAP patients, the results showed that the infection rate, the incidence of multiple organ dysfunction syndrome, and the length of hospital stay were significantly reduced, and that rhubarb combined with early EN improves the intestinal function and inhibits systemic inflammation, which is a significant therapeutic effect for SAP patients (Chen et al., 2020).

### 3.5 Traditional Chinese medicine

Chinese medicine has unique advantages in the treatment of SAP. Picroside II, with its antioxidant and anti-inflammatory activities, improved the intestinal microbiota of SAP rats after Picroside II treatment, as evidenced by the enrichment of the abundance of *Lactobacillus* and *Prevotella*, and the decrease in the abundance of *Helicobacter* and *Escherichia Shigella*, which resulted in the improvement of the intestinal barrier damage of SAP, and the decrease of the occurrence of infectious complications (Piao et al., 2020). Saikosaponin A likewise functions to improve the composition of the gut microbiota, thereby further reducing the systemic inflammatory response to SAP (Li et al., 2020c). Zhang et al. found that resveratrol pre-treatment attenuated pancreatic injury, inflammation and oxidative stress in hypertriglyceridemic acute pancreatitis (HTG-AP) mice by mechanisms related to inhibition of the NF-κB signalling pathway and modulation of the gut microbiota (Zhang et al., 2022). Qingyi Granules and its main active ingredient emodin treat SAP by restoring intestinal microbiota homeostasis to maintain intestinal barrier integrity and inhibit systemic inflammatory responses (Jiao et al., 2023).

## 4 Gut microbiota-derived extracellular vesicles in the treatment of acute pancreatitis

Research on the therapeutic potential of extracellular vesicles has received attention in recent years. One study found that acinar cell-derived exosomes reduced acinar cell apoptosis and ROS production, and may alleviate the severity of AP through the MAPK and NF-κB signalling pathways (Guo et al., 2021). Chen et al. showed that exosome from human induced pluripotent stem cells derived mesenchymal stem cells (iMSCs) can alleviate SAP-induced myocardial injury (MI) by activating the Nrf2/HO-1 axis (Chen et al., 2022). Exosomes from adipose-derived mesenchymal stem cells (AdMSC-Exos) can supply mitochondrial components, improve mitochondrial integrity and function in macrophages, and increase the level of oxidative phosphorylation, thereby restoring metabolic and immune homeostasis in macrophages in the airway and alleviating ALI (Xia et al., 2022). Therefore, the regulatory role of extracellular vesicles provides a new idea for the treatment of AP and its complications, which has a certain reference value. After the occurrence of AP, the intestinal permeability increases, the intestinal flora is disordered, and bacterial translocation is likely to occur, resulting in “secondary infection,” so maintaining the structural and functional integrity of the intestinal barrier plays an important role in the prevention of local or systemic infectious complications of AP (Cen et al., 2018a). Extracellular Vesicles Derived from Kefir Grain *Lactobacillus* may exert anti-inflammatory effects by regulation of phosphorylation/degradation of IκBα in NF-κB signaling pathway, preventing the disruption of tight junction structures and tight junction-associated proteins, thereby reducing intestinal permeability and protecting the intestinal barrier (Kang et al., 2020). Additional finding suggested that the extracellular vesicles from *L. plantarum Q7* could increase beneficial bacteria and decrease harmful bacteria in the gut, improving intestinal barrier function (Hao et al., 2021). *Lactobacillus rhamnosus GG* derived extracellular vesicles similarly inhibited the production of pro-inflammatory factors and restored the homeostasis of the intestinal flora (Tong et al., 2021). These findings provide new perspectives to explore the mechanism of action of gut microbiota-derived EVs and to develop potential treatments for AP and its complications. In the future we can try to widen the use of gut microbiota-derived EVs in AP and its complications.

## 5 Conclusions and perspectives

During AP, increased intestinal permeability and disturbances in the intestinal flora predispose to bacterial translocation, which can lead to the development of local or systemic complications, thus we cannot ignore the serious consequences of AP. Recent studies have shown that there is a link between pancreatitis and intestinal microbiota, and researchers have started to regulate the intestinal flora to improve the complications of AP. This is accompanied by the emergence of extracellular vesicles, which provides new therapeutic ideas for the treatment of AP and its complications.

In conclusion, the significance of AP and the gut microbiome is gradually being appreciated by researchers, which opens up new horizons for in-depth studies of AP. Imbalance of the gut microbiota is responsible for the disruption of intestinal homeostasis and the development of complications during AP. However, the link between AP and gut flora dysbiosis is not fully understood and needs to be further elucidated. Based on current studies, the effectiveness of treating AP complications based on modulating gut flora and utilising gut microbiota-derived EVs has not been clarified. Therefore, large-scale, multilevel trials are needed to further explore research on probiotics and prebiotics, FMT, and gut microbiota-derived EVs for the treatment of AP complications.
