# Supplementary material for: Exploring the gut microbiota’s crucial role in acute pancreatitis and the novel therapeutic potential of derived extracellular vesicles
Source: Front Pharmacol. 2024 Jul 26;15:1437894. doi: 10.3389/fphar.2024.1437894 (PMC11310017; doi:10.3389/fphar.2024.1437894)
Supplement: Supplementary file 1 [file Table1.docx]

Supplementary Material

# Supplementary Tables

**Table 1 Altered microbiome composition observed in acute pancreatitis.**

| No. | Type of species | Type of sample | Techniques used for microbiome profiling | AP | MAP | MSAP | SAP | Ref. |
| --- | --- | --- | --- | --- | --- | --- | --- | --- |
| 1 | Rats | Feces | 16S rRNA sequencing | *Clostridiaceae 1*⬆ *Clostridium sensu stricto 1*⬆ | *Bacteroidales S24-7*⬆  *Bacteroidales S24-7 group norank*⬆ | NA | *Collinsella⬆ Morganella⬆ Blautia⬆*  *Lactobacillus*⬇ | (Liu et al., 2022) |
| 2 | Human | Feces | 16S rRNA sequencing | *Enterobacteriales*⬆  *Clostridiales* ⬇  *Bacteroidales*⬇ | NA | NA | NA | (Zou et al., 2022) |
| 3 | Mice | Feces | 16S rRNA sequencing | *Butyrate-producing bacteria*⬇  *Akkermansia muciniphila*⬆ | NA | NA | NA | (F F Van Den Berg et al., 2021) |
| 4 | Rats | Feces | qPCR | NA | NA | NA | *Escherichia*⬆  *Lactobacillus*⬇  *Bifidobacterium*⬇ | (Su and Tang, 2021) |
| 5 | Human | Feces | 16S rRNA sequencing | *Escherichia-Shigella*⬆ *Enterococcus*⬆  *Dorea longicatena*⬇  *Blautia wexlerae*⬇  *Bacteroides Ovatus*⬇ | NA | NA | NA | (Hu et al., 2021) |
| 6 | Mice | Feces | 16S rRNA sequencing | NA | NA | NA | *Escherichia-Shigella*⬆  *Enterococcus*⬆  *Akkermansia*⬇ | (Mei et al., 2021) |
| 7 | Human | Rectal swab | shotgun metagenomic sequencing | NA | *Streptococcus*⬇ | *Escherichia-coli*⬇ | *Enterococcus*⬇ | (Yu et al., 2021) |
| 8 | Rats | Feces | 16S rRNA sequencing | NA | NA | NA | *Helicobacter*⬆  *Escherichia-Shigella*⬆  *Lactobacillus*⬇  *Prevotella*⬇ | (Piao et al., 2020a) |
| 9 | Human | Rectal swab | 16S rRNA sequencing | NA | *Bacteroides*⬆  *Finegoldia*⬆  *Blautia*⬇ | *Escherichis-Shigella*⬆  *Anaerococcus*⬆  *Eubacterium hallii*⬇ | *Enterococcus*⬆  *Eubacterium hallii*⬇ | (Yu et al., 2020) |
| 10 | Mice | Feces | 16S rRNA sequencing | *Proteobacteria*⬆  *Escherichia/Shigella*⬆  *Streptococcus*⬆ | NA | NA | NA | (Fons F Van Den Berg et al., 2021) |
| 11 | Mice | Feces | 16S rRNA sequencing | *Escherichia-Shigella*⬆  *Enterococcus*⬆  an unclassified member in *Enterococcaceae*⬆  *Blautia*⬇  Members in *Lachnospiraceae*⬇  Members in *Ruminococcaceae*⬇ | NA | NA | *Acinetobacter*⬆ *Stenotrophomonas*⬆ *Geobacillus*⬆  *Bacteroides*⬇ *Alloprevotella*⬇ *Blautia*⬇  *Gemella*⬇ | (Zhu et al., 2019) |
| 12 | Human | Blood | 16S rDNA-based next-generation sequencing | NA | NA | NA | *Bacteroides*⬆  *Stenotrophomonas*⬆  *Serratia*⬆  *Rhizobium*⬆  *Prevotella*⬆  *Staphylococcus*⬆  *Paracoccus*⬆  *Acinetobacter*⬇  *Lactococcus*⬇  *Flavobacterium*⬇  *Pseudomonas*⬇  *Corynebacterium*⬇  Sphingobium⬇ | (Li et al., 2018) |
| 13 | Human | Feces | 16S rRNA sequencing | *Bacteroidetes*⬆ *Proteobacteria*⬆  *Firmicutes*⬇ *Actinobacteria*⬇ | NA | NA | NA | (Zhang et al., 2018) |
| 14 | Rats | Feces | 16S rRNA sequencing | *Saccharibacteria*⬇  *Tenericutes*⬇  *Escherichia-Shigella*⬆ *Phascolarctobacterium*⬆  *Candidatus Saccharimonas*⬇  *Prevotellaceae UCG-001*⬇  *Lachnospiraceae UCG-001*⬇  *Ruminiclostridium 5*⬇ *Ruminococcaceae UCG-008*⬇ | NA | NA | NA | (Chen et al., 2017) |
| 15 | Human | Feces | qPCR  (performed only on MAP and SAP patients) | NA | *Enterobacteriaceae*⬆  *Enterococci*⬆  *Bifidobacteria*⬇ | NA | *Enterobacteriaceae*⬆  *Enterococci*⬆  *Bifidobacteria*⬇ | (Tan et al., 2015) |

MAP: Mild acute pancreatitis; MSAP: Moderately severe acute pancreatitis; NA: Not available;

SAP: Severe acute pancreatitis; ⬆: Higher level; ⬇: Lower level.

**Table 2 The therapeutic methods and research advancements in modulating gut microbiota for treating AP and its complications.**

| **Treatment Methods** | **Functions** | **Research Progress** | **Challenges and Prospects** | **Ref.** |
| --- | --- | --- | --- | --- |
| Probiotics and prebiotics | Regulate intestinal flora balance;  Enhance intestinal barrier function;  Suppress inflammatory response | Multiple clinical trials have demonstrated that probiotics can improve the clinical symptoms of patients with acute pancreatitis. | More precise identification of the species and dose of probiotics and the long-term effects and safety require further verification. | (Wu et al., 2020)  (Wan et al., 2021)  (Li et al., 2022)  (Zhai et al., 2018)  (Wang et al., 2023)  (Sharma et al., 2011) |
| Fecal Microbiota Transplantation | Rebuilding a healthy intestinal flora;  Restoring intestinal microecological balance | Preliminary clinical trials and animal model studies have shown that fecal microbiota transplantation has potential benefits for patients with acute pancreatitis. | More studies are needed to verify its safety and efficacy;  The stability of intestinal flora after transplantation needs further observation. | (Tang et al., 2021)  (Ding et al., 2022) |
| Antibiotics | Suppress the growth of harmful bacteria;  Reduce complications of bacterial infections | The results of clinical trials are controversial, and some studies have not shown significant benefits. | The issue of antibiotic resistance；  It may disrupt the intestinal microbial balance and increase the risk of other complications. | (Soares et al., 2017)  (Leppäniemi et al., 2019)  (Wittekamp et al., 2020) |
| Enteral Nutrition | Regulate intestinal flora balance;  Restoring intestinal barrier function；  Reduce complications of bacterial infections | Preliminary studies suggest that EN may help improve the intestinal flora and symptoms of patients with acute pancreatitis, reducing the occurrence of complications. | More specific and personalized enteral nutrition recommendations are needed, and the long-term effects require further observation. | (Al-Omran et al., 2010)  (Liu et al., 2023)  (Qin et al., 2008)  (Wang et al., 2013)  (Chen et al., 2020) |
| Traditional Chinese Medicine | Regulate intestinal flora balance;  Suppress inflammatory response | Preliminary research explores the potential of Traditional Chinese Medicine in the treatment of acute pancreatitis. | More basic and clinical research is needed for verification. | (Piao et al., 2020b)  (Li et al., 2020)  (Zhang et al., 2022)  (Jiao et al., 2023) |
